# Supplementary material for: Status, Sources and Assessment of Potentially Toxic Element (PTE) Contamination in Roadside Orchard Soils of Gaziantep (Türkiye)
Source: Int J Environ Res Public Health. 2023 Jan 30;20(3):2467. doi: 10.3390/ijerph20032467 (PMC9916285; doi:10.3390/ijerph20032467)
Supplement: Supplementary file 1 [file ijerph-20-02467-s001.zip › ijerph-2150984-supplementary.pdf]

International Journal of
Environmental Research and Public Health

MDPI

Status, Sources and Assessment of Potentially Toxic Element (PTE) Contamination in Roadside Orchard Soils of Gaziantep (Türkiye)

Mustafa Demir [1], Erdihan Tunç [1,*], Sören Thiele-Bruhn [2], Ömer Çelik [3], Awet Tekeste Tsegai [4], Nevzat Aslan [5] and Sevgi Arslan [1]

Supplementary Materials

Table S1. The traffic density of roads in the last ten years [36].

	Year	GAH (D400)	GKH (D850)	GNH (D400)	GYH (D850)
The traffic density (vehicle/day)	2003	4,410	2,294	10,614	2,187
	2004	4,664	2,545	11,543	2,336
	2005	4,280	2,879	12,053	2,510
	2006	4,466	3,079	9,919	2,287
	2007	4,339	3,247	9,585	2,380
	2008	4,436	3,295	8,617	2,352
	2009	4,678	3,479	8,818	3,256
	2010	5,169	5,119	9,301	2,599
	2011	5,627	4,933	10,555	3,267
	2012	6,398	6,128	11,451	4,176
	2013	6,176	7,530	11,393	5,352
	2014	7,230	7,893	13,018	6,251
	2015	7,637	7,980	12,890	6,780
	2016	8,149	8,125	13,979	7,490
	2017	8,554	8,771	15,277	7,935
	2018	8,012	9,258	14,994	7,906
	2019	4,410	2,294	10,614	2,187
Mean (vehicle.day^{-1})		5,889	5,410	11,500	4,317
Mean (vehicle.h^{-1})		245	225	479	180
Classification		MTD	MTD	HTD	LTD

Low traffic density (LTD) < 200 vehicles.h^{-1}

200 vehicles.h^{-1} < Medium traffic density (MTD) < 400 vehicles.h^{-1}

400 vehicles.h^{-1} < High traffic density (HTD)

Table S2. Certificate values and measured values of reference material (UME EnvCRM 03, TÜ-BİTAK) and limits of quantification (LOQ) and detection (LOD) for metals and PTEs.

	Absorbance (nm)	Certified values (mg.kg^{-1})	Measured values (mg.kg^{-1})		Coverage (%)	LOQ (mg.kg^{-1})	LOD (mg.kg^{-1})
			Mean	± SD			
**Cd**	214.439	1.29	1.30	± 0.04	100.62	0.0012	0.0040
**Co**	228.615	42	41.82	± 0.31	99.57	0.0022	0.0073
**Cr**	267.716	115.6	115.2	± 1.13	99.65	0.0018	0.0060
**Cu**	324.754	63.5	63.68	± 0.31	100.28	0.0014	0.0047
**Fe**	234.350	26,748	26,784	± 177	100.14	0.0016	0.0053
**Mn**	260.568	674	673	± 3.67	99.85	0.0018	0.0060
**Ni**	231.604	51.7	51.96	± 0.50	100.50	0.0022	0.0073
**Pb**	283.305	64.1	64.36	± 0.93	100.41	0.0013	0.0043
**Zn**	213.857	150.6	150.44	± 1.48	99.89	0.0016	0.0053

Table S3. The ranges and classifications of individually environmental and ecological indexes.

	Indexes	Cls.	Range	Classification	Reference
Complex Indexes	Modified degree of contamination (*mCd*)	1	$mCd < 1.5$	Very low contamination	[44]
		2	$1.5 \leq mCd < 2$	Low contamination	
		3	$2 \leq mCd < 4$	Moderate contamination	
		4	$4 \leq mCd < 8$	High contamination	
		5	$8 \leq mCd < 16$	Very high contamination	
		6	$16 \leq mCd < 32$	Extremely high contamination	
		7	$32 \leq mCd$	Ultra high contamination	
	The potential ecological risk index (*RI*)	1	$RI < 50$	Low ecological risk	[43]
		2	$50 \leq RI < 100$	Moderate ecological risk	
		3	$100 \leq RI < 200$	Considerable ecological risk	
		4	$200 \leq RI$	Very high ecological risk	
	Average of pollution index (*PIave*)	1	$PIave< 1$		[31,45]
		2	$1 \leq PI_{ave}$	Low quality soil because of contamination	
Individually Indexes	The contamination factor (*Cf*)	1	$Cf < 1$	Low contamination	[43]
		2	$1 \leq Cf < 3$	Moderate contamination	
		3	$3 \leq Cf < 6$	Considerable contamination	
		4	$6 \leq Cf$	Very high contamination	
	The potential ecological risk factor (*ER*)	1	$ER < 40$	Low potential ecological risk	[43]
		2	$40 \leq ER < 80$	Moderate potential ecological risk	
		3	$80 \leq ER < 160$	Considerable potential ecological risk	
		4	$160 \leq ER < 320$	High potential ecological risk	
		5	$320 \leq ER$	Very high potential ecological risk	
	The single pollution index (*PI*)	1	$PI < 1$	Absent	[31,45]
		2	$1 \leq PI < 2$	Low pollution	
		3	$2 \leq PI < 3$	Moderately pollution	
		4	$3 \leq PI < 5$	Strong pollution	
		5	$5 \leq PI$	Very strong pollution	
	The enrichment factor (*EF*)	1	$EF < 2$	Deficiency to minimal enrichment	[48]
		2	$2 \leq EF < 5$	Moderate enrichment	
		3	$5 \leq EF < 20$	Significant enrichment	
		4	$20 \leq EF < 40$	Very high enrichment	
		5	$40 \leq EF$	Extremely enrichment	
	The soil pollution index (*SOPI*)	1	$SOPI < 1.5$	No pollution	In this study
		2	$1.5 \leq SOPI < 2$	Low pollution	
		3	$2 \leq SOPI < 3$	Moderately pollution	
		4	$3 \leq SOPI < 4$	Significantly pollution	
		5	$4 \leq SOPI < 5$	Highly pollution	
		6	$5 \leq SOPI$	Extremely pollution	

Table S4. Toxicity response factor and pre-industrial reference level ($mg.kg^{-1}$) values.

	**Cd**	**Cr**	**Cu**	**Ni**	**Pb**	**Zn**	**Ref.**
Toxicity response factor (*TR*)	30	2	5	5	5	1	[31,43]
Pre-industrial reference level (*PIV*)	1.00	90.00	50.00	5.00	70.00	175.00	

Table S5. Values of complex and individually index (Letters a, b, c: indicate significant differences at $p < 0.05$).

			DtR						TrD				
			10 m	25 m	50 m	100 m	r^2	*p*-Value	LTD	MTD	HTD	r^2	*p*-Value
Complex Indexes		*mCd*	3.85^{a}	3.79^{a}	3.67^{a}	3.86^{a}	-0.007	0.995	5.39^{c}	3.81^{b}	2.15^{a}	**-,796****	**0.000**
		RI	119.38^{a}	117.35^{a}	114.33^{a}	119.19^{a}	-0.010	0.995	167.19^{c}	116.96^{b}	69.14^{a}	**-,829****	**0.000**
		PIave	2.17^{a}	2.15^{a}	2.13^{a}	2.14^{a}	-0.032	0.998	2.69^{c}	2.14^{b}	1.60^{a}	**-,845****	**0.000**
Individually Indexes — According to PIV [31,43]	*Cf*	Cd	0.30^{a}	0.30^{a}	0.31^{a}	0.28^{a}	-0.041	0.977	0.37^{a}	0.26^{a}	0.30^{a}	-0.223	0.108
		Cr	0.83^{a}	0.82^{a}	0.80^{a}	0.81^{a}	-0.036	0.995	0.97^{b}	0.89^{b}	0.50^{a}	**-.602****	**0.000**
		Cu	0.36^{a}	0.35^{a}	0.35^{a}	0.36a	0.011	0.995	0.40^{b}	0.38^{b}	0.27^{a}	**-.698****	**0.000**
		Ni	20.37^{a}	20.01^{a}	19.33^{a}	20.45^{a}	-0.006	0.994	29.34^{c}	20.06^{b}	10.71^{a}	**-.799****	**0.000**
		Pb	0.87^{a}	0.86^{a}	0.86^{a}	0.87^{a}	-0.004	0.999	0.93^{b}	0.88^{b}	0.76^{a}	**-.562****	**0.002**
		Zn	0.37^{a}	0.38^{a}	0.38^{a}	0.36^{a}	-0.024	0.957	0.33^{a}	0.40^{b}	0.37^{ab}	0.185	0.091
	ER	Cd	9.05^{a}	8.86^{a}	9.30^{a}	8.47^{a}	-0.041	0.977	11.14^{a}	7.83^{a}	8.88^{a}	-0.223	0.108
		Cr	1.67^{a}	1.63^{a}	1.59^{a}	1.62^{a}	-0.036	0.995	1.95^{b}	1.79^{b}	1.00^{a}	**-.602****	**0.000**
		Cu	2.14^{a}	2.10^{a}	2.12^{a}	2.15^{a}	0.011	0.995	2.38^{b}	2.27^{b}	1.60^{a}	**-.698****	**0.000**
		Ni	101.83^{a}	100.07^{a}	96.65^{a}	102.27^{a}	-0.006	0.994	146.72^{c}	100.28^{b}	53.53^{a}	**-.799****	**0.000**
		Pb	4.33^{a}	4.30^{a}	4.29^{a}	4.33^{a}	-0.004	0.999	4.67^{b}	4.40^{b}	3.78^{a}	**-.562****	**0.002**
		Zn	0.37^{a}	0.38^{a}	0.38^{a}	0.36^{a}	-0.024	0.957	0.33^{a}	0.40^{b}	0.37^{ab}	0.185	0.091
Individually Indexes — According to UCC [31,45,47]	*PI*	Cd	2.98^{a}	2.90^{a}	3.04^{a}	2.77^{a}	-0.041	0.977	3.64^{a}	2.56^{a}	2.90^{a}	-0.223	0.108
		Co	1.30^{a}	1.31^{a}	1.26^{a}	1.28^{a}	-0.028	0.997	1.93^{c}	1.19^{b}	0.85^{a}	**-.914****	**0.000**
		Cr	2.15^{a}	2.10^{a}	2.04^{a}	2.09^{a}	-0.036	0.995	2.50^{b}	2.30^{b}	1.28^{a}	**-.602****	**0.000**
		Cu	1.25^{a}	1.22^{a}	1.24^{a}	1.25^{a}	0.011	0.995	1.39^{b}	1.32^{b}	0.93^{a}	**-.698****	**0.000**
		Fe	0.77^{a}	0.76^{a}	0.77^{a}	0.78^{a}	0.032	0.992	0.89^{c}	0.81^{b}	0.57^{a}	**-.818****	**0.000**
		Mn	0.80^{a}	0.85^{a}	0.78^{a}	0.77^{a}	-0.074	0.851	1.04^{c}	0.78^{b}	0.59^{a}	**-.851****	**0.000**
		Ni	5.47^{a}	5.38^{a}	5.20^{a}	5.50^{a}	-0.006	0.994	7.89^{c}	5.39^{b}	2.88^{a}	**-.799****	**0.000**
		Pb	3.57^{a}	3.54^{a}	3.54^{a}	3.56^{a}	-0.004	0.999	3.84^{b}	3.62^{b}	3.11^{a}	**-.562****	**0.002**
		Zn	1.23^{a}	1.28^{a}	1.27^{a}	1.22^{a}	-0.024	0.957	1.11^{a}	1.33^{b}	1.23^{ab}	0.185	0.091
	EF	Cd	3.97^{a}	4.01^{a}	4.14^{a}	3.71^{a}	-0.046	0.967	4.10^{ab}	3.33^{a}	5.07^{b}	0.210	0.047
		Co	1.65^{a}	1.71^{a}	1.63^{a}	1.62^{a}	-0.063	0.952	2.18^{b}	1.47^{a}	1.49^{a}	**-.751****	**0.000**
		Cr	2.71^{a}	2.70^{a}	2.61^{a}	2.62^{a}	-0.076	0.976	2.82^{b}	2.79^{b}	2.26^{a}	**-.374***	**0.042**
		Cu	1.63^{a}	1.62^{a}	1.61^{a}	1.61^{a}	-0.098	0.952	1.56^{a}	1.63^{b}	1.64^{b}	**.434***	**0.013**
		Fe	1.00^{a}	1.00^{a}	1.00^{a}	1.00^{a}	0.000	1.000	1.00^{a}	1.00^{a}	1.00^{a}	0.000	1.000
		Mn	1.03^{a}	1.12^{a}	1.02^{a}	1.00^{a}	-0.136	0.363	1.18^{b}	0.97^{a}	1.05^{a}	-0.331	0.002
		Ni	6.81^{a}	6.85^{a}	6.56^{a}	6.81^{a}	-0.018	0.990	8.88^{c}	6.54^{b}	5.07^{a}	**-.739****	**0.000**
		Pb	4.77^{a}	4.76^{a}	4.66^{a}	4.63^{a}	-0.100	0.958	4.32^{a}	4.50^{a}	5.51^{b}	**.712****	**0.000**
		Zn	1.67^{a}	1.74^{a}	1.69^{a}	1.60^{a}	-0.076	0.924	1.25^{a}	1.65^{b}	2.16^{c}	**.789****	**0.000**
	SOPI	Cd	1.94^{a}	1.91^{a}	1.95^{a}	1.83^{a}	-0.063	0.965	1.94^{ab}	1.74^{a}	2.22^{b}	0.209	0.058
		Co	1.27^{a}	1.26^{a}	1.26^{a}	1.27^{a}	0.001	0.995	1.42^{b}	1.22^{a}	1.21^{a}	**-.815****	**0.000**
		Cr	1.63^{a}	1.60^{a}	1.60^{a}	1.61^{a}	-0.028	0.985	1.61^{ab}	1.67^{b}	1.48^{a}	**-0.267**	**0.038**
		Cu	1.27^{a}	1.24^{a}	1.26^{a}	1.27^{a}	0.046	0.788	1.20^{a}	1.29^{b}	1.27^{b}	**.447***	**0.000**
		Fe	0.99^{a}	0.98^{a}	1.00^{a}	1.00^{a}	0.135	0.456	0.96^{a}	1.01^{a}	0.99^{a}	0.311	0.001
		Mn	1.01^{a}	1.02^{a}	1.00^{a}	1.00^{a}	-0.135	0.456	1.04^{a}	0.99^{a}	1.01^{a}	-0.311	0.001
		Ni	2.57^{a}	2.52^{a}	2.53^{a}	2.59^{a}	0.022	0.980	2.87^{c}	2.56^{b}	2.23^{a}	**-.667****	**0.000**
		Pb	2.16^{a}	2.13^{a}	2.15^{a}	2.15^{a}	-0.020	0.979	2.00^{a}	2.14^{b}	2.32^{c}	**.758****	**0.000**
		Zn	1.28^{a}	1.28^{a}	1.29^{a}	1.26^{a}	-0.033	0.988	1.08^{a}	1.29^{b}	1.45^{c}	**.799****	**0.000**
